# Case Report: Relapsed acquired hemophilia A following prednisone-rituximab therapy successfully treated with orelabrutinib

**DOI:** 10.3389/fimmu.2026.1931049

**Published:** 2026-10-28

**Authors:** Yujie Guo, Feifei Zhang, Yabei Zuo, Yan Wang, Zhiyun Niu, Jingyu Zhang

**Affiliations:** 1 Department of Hematology, The Second Hospital of Hebei Medical University, Hebei Key Laboratory of Hematology, Shijiazhuang, China; 2 Special Examination Department, The Second Hospital of Hebei Medical University, Shijiazhuang, China

**Keywords:** acquired hemophilia A, autoantibody, Bruton’s tyrosine kinase, BTK inhibitor, relapse

## Abstract

**Introduction:**

Acquired hemophilia A (AHA) is a rare autoimmune hemorrhagic disorder characterized by neutralizing autoantibodies against coagulation factor VIII (FVIII). First-line immunosuppressive therapy with corticosteroids with or without rituximab achieves complete remission (CR) in 60-80% of patients; however, relapse occurs in approximately 15-33% of cases after treatment cessation. Management of relapsed AHA remains challenging, with limited evidence for optimal second-line approaches. Bruton’s tyrosine kinase (BTK) inhibitors have emerged as promising agents for antibody-mediated autoimmune diseases.

**Case description:**

Herein, we report the first case of relapsed AHA successfully treated with orelabrutinib monotherapy after relapse following an initial response to a conventional corticosteroid-rituximab regimen. A 45-year-old male with type 1 diabetes mellitus and hyperthyroidism presented with spontaneous bleeding and was diagnosed with AHA associated with underlying autoimmune endocrinopathies. He achieved complete remission (CR) following prednisone and rituximab but relapsed 3 months after treatment cessation. Orelabrutinib 50 mg orally once daily was initiated as an experimental off-label approach. The patient demonstrated progressive laboratory improvement, achieving a second CR approximately 2 months after orelabrutinib initiation, with undetectable FVIII inhibitor and normalized FVIII activity. Sustained on-treatment remission was maintained throughout 5 months of therapy without bleeding episodes or adverse events, and remission has been maintained after completion of the planned 6-month course and subsequent treatment discontinuation.

**Conclusion:**

This case supports the therapeutic potential of selective BTK inhibition as a targeted, steroid-sparing strategy in relapsed AHA and highlights the need for prospective clinical trials to validate this approach.

## 1 Introduction

Acquired hemophilia A (AHA) is a rare but life-threatening autoimmune hemorrhagic disorder with an estimated incidence of 1-1.5 per million per year (1). The condition is characterized by the development of neutralizing autoantibodies (inhibitors) directed against coagulation factor VIII (FVIII), resulting in prolonged activated partial thromboplastin time (APTT), reduced FVIII activity, and spontaneous bleeding manifestations (2, 3). Unlike congenital hemophilia, AHA typically affects elderly individuals without prior bleeding history, with approximately 50% of cases associated with underlying autoimmune conditions, malignancies, pregnancy, or drug exposure (4).

First-line immunosuppressive therapy with corticosteroids alone or in combination with rituximab achieves complete remission (CR) in 60-80% of patients; however, relapse occurs in approximately 15-33% of cases after treatment cessation (3–5). The pathophysiology of relapse likely involves incomplete depletion of autoreactive B-cell clones and persistence of long-lived plasma cells secreting anti-FVIII antibodies (1). Management of relapsed AHA presents a significant clinical challenge, as re-treatment with corticosteroids carries cumulative toxicity risks, particularly in elderly patients with comorbidities (1, 6). Rituximab re-exposure may be less effective due to residual B-cell depletion or development of resistance. Alternative agents including cyclophosphamide, bortezomib, or calcineurin inhibitors have been explored in refractory cases, but evidence remains limited to small case series (6, 7).

Bruton’s tyrosine kinase (BTK) is a critical mediator of B-cell receptor (BCR) signaling, regulating B-cell development, activation, proliferation, and survival (8). BTK inhibitors have emerged as promising targeted agents for antibody-mediated hematologic disorders and autoimmune diseases by specifically interrupting BCR-dependent signaling pathways (9, 10). First-generation BTK inhibitors such as ibrutinib have demonstrated efficacy in immune thrombocytopenia (ITP), autoimmune hemolytic anemia, and acquired hemophilia associated with B-cell lymphoproliferative disorders (11–14).

Orelabrutinib is a highly selective, covalent BTK inhibitor with improved kinase selectivity compared to first-generation agents such as ibrutinib, minimizing off-target effects on epidermal growth factor receptor (EGFR), interleukin-2-inducible T-cell kinase (ITK), and Tec family kinases (15). This enhanced selectivity translates to a reduced incidence of toxicities including atrial fibrillation, bleeding, and diarrhea. While BTK inhibitors have demonstrated efficacy in ITP (16, 17) and autoimmune hemolytic anemia (18), their application in acquired hemophilia remains largely unexplored. Herein, we report the first case of relapsed AHA successfully treated with orelabrutinib monotherapy after relapse following an initial response to a conventional corticosteroid-rituximab regimen.

## 2 Case description

A 45-year-old male with type 1 diabetes mellitus and hyperthyroidism presented in June 2025 with spontaneous bleeding for two weeks, including left toe hemorrhage, left forearm swelling, and acute left knee joint swelling with pain. Physical examination revealed scattered ecchymoses, left knee swelling with tenderness, left lower leg edema, and extensive foot ecchymosis.

Coagulation studies showed markedly prolonged activated partial thromboplastin time (APTT) at 61.90 s (normal range 25.40-38.40 s) with normal prothrombin time. The APTT mixing study demonstrated immediate near correction to 39.40 s with prolongation to 50.70 s after 2-hour incubation at 37 degrees C, indicating a time-dependent inhibitor pattern. FVIII activity was severely reduced at 4.90% (normal 50-150%), with an inhibitor titer of 73.60 Bethesda units (BU) measured by the Nijmegen-modified Bethesda assay. Autoimmune disease-related antibody screening results were negative. The patient had no personal history of abnormal bleeding, including after minor trauma, surgery, or dental procedures, and had never been exposed to FVIII concentrates or other blood products; together with the adult onset of symptoms and the time- and temperature-dependent inhibitor pattern, these findings excluded congenital hemophilia A. Given his coexisting autoimmune endocrinopathies (type 1 diabetes mellitus and hyperthyroidism), the case was classified as AHA associated with underlying autoimmune disorders rather than strictly idiopathic. Notably, his younger brother had previously been diagnosed with AHA, achieved CR, and remained relapse-free. Familial clustering of AHA is exceedingly rare; a shared genetic predisposition remains a hypothesis that warrants dedicated genetic studies.

On June 26, 2025, the patient commenced first-line immunosuppressive therapy comprising prednisone 70 mg/day (1 mg/kg/day) orally and rituximab 375 mg/m2 intravenous weekly for 4 cycles. Concurrently, prothrombin complex concentrate was administered at a dose of 30 IU/kg every 12 hours for bypass hemostasis during the initial treatment phase, for a total course of 5 days. Treatment response was monitored serially as shown in **Table 1**.

**Table 1:** Laboratory parameters and clinical response during first-line immunosuppressive therapy with prednisone and rituximab.

Date	Jun 25 2025	Jun 26 2025	Jul 3 2025	Jul 11 2025	Jul 18 2025	Aug 14 2025	Normal range
APTT (s)	61.90	–	53.60	33.20	33.20	34.50	25.40-38.40
FVIII:C (%)	4.90	–	8.90	17.50	50.40	55.60	50.00-150.00
FVIII Inhibitor (BU)	73.60	–	4.80	<0.60	<0.60	<0.60	<0.60
Clinical Status	Diagnosis intiated	Treatment initiated	–	Inhibitor depleted	CR achieved	Continued CR	–

By July 18, 2025 (approximately 3 weeks after treatment initiation), the patient achieved CR, defined according to international recommendations as FVIII activity of at least 50% with an undetectable inhibitor (<0.60 BU) (2): (1) APTT normalization; (2) FVIII:C recovery to 50.4%; (3) undetectable FVIII inhibitor (<0.60 BU); and (4) absence of bleeding manifestations. Because this remission was achieved while corticosteroids were still being tapered, it represents an on-treatment remission rather than a treatment-free CR. Corticosteroids were subsequently tapered and discontinued over the following one month. FVIII:C remained at 55.6% with undetectable inhibitor on August 14, 2025.

After approximately 3 months of immunosuppression-free follow-up, the patient presented with recurrent gingival bleeding for one week on November 13, 2025. Laboratory evaluation confirmed relapse: although the APTT (36.00 s) remained within the reference range (25.40-38.40 s), FVIII:C had declined to 50.00% from its post-treatment peak, and the FVIII inhibitor (Nijmegen-modified Bethesda assay) had re-emerged at 2.20 BU on November 13, 2025, rising to 3.20 BU on November 19, 2025. The low-titer positivity was corroborated by this rising titer on serial measurements. To exclude analytical interference, supplementary testing was performed: serum protein electrophoresis with immunofixation revealed no monoclonal protein, and lupus anticoagulant testing (dilute Russell’s viper venom time and silica clotting time) was negative. The diagnosis of AHA relapse was established. Given the prior exposure to corticosteroids and rituximab, and concerns regarding repeated high-dose corticosteroid toxicity (particularly in a patient with type 1 diabetes), alternative therapeutic strategies were considered.

After multidisciplinary discussion and informed consent, the patient was initiated on orelabrutinib monotherapy 50 mg orally once daily on November 19, 2025, as an experimental off-label approach for relapsed AHA. This decision was based on (1): the established role of BTK inhibitors in antibody-mediated autoimmune disorders (2); the need for steroid-sparing therapy given comorbidities (3); the favorable safety profile of orelabrutinib compared to non-selective BTK inhibitors; and (4) the absence of approved second-line AHA therapies with demonstrated superiority over first-line re-treatment. The 50 mg once-daily dose was selected on the basis of the phase 2 trial of orelabrutinib in immune thrombocytopenia, in which this dose demonstrated efficacy with a favorable safety profile (17); given the patient’s active bleeding diathesis, the higher dose approved for B-cell malignancies was considered unnecessary for immune modulation. The planned 6-month treatment duration was extrapolated from international AHA recommendations, which advise maintaining immunosuppression until sustained inhibitor eradication, typically for several months after complete remission (2, 3), and from treatment durations reported for BTK inhibitors in autoimmune cytopenias (17, 19); we acknowledge that the optimal duration remains empirical.

The patient demonstrated progressive laboratory improvement during orelabrutinib therapy, as summarized in **Table 2**. On December 9, 2025, APTT was 33.80 s, FVIII:C 57.70%, and FVIII inhibitor 1.40 BU, indicating early response. By January 19, 2026, approximately 2 months after orelabrutinib initiation, the inhibitor became undetectable (<0.60 BU) with FVIII:C at 57.70%, achieving a second CR (CR2); like CR1, CR2 represents an on-treatment remission. At the most recent follow-up on May 7, 2026 (approximately 5 months of orelabrutinib therapy), the patient demonstrated: APTT 30.2 s (within normal range), FVIII:C 83.1%, and undetectable FVIII inhibitor (**Figure 1**). Clinically, no bleeding episodes occurred since orelabrutinib initiation, and gingival bleeding completely resolved. Adverse events were monitored prospectively through weekly clinical assessments, complete blood counts every 2 weeks, liver function tests every 4 weeks, and electrocardiography at months 1, 3, and 6. No adverse events, including bleeding, infections, cytopenias, hepatotoxicity, diarrhea, or cardiovascular events, were observed. Orelabrutinib was discontinued on May 19, 2026, after completion of the planned 6-month course. At the most recent follow-up, approximately 3 months after discontinuation, the patient remained in treatment-free complete remission, with FVIII activity within the normal range and an undetectable FVIII inhibitor.

**Table 2:** Laboratory parameters and clinical response during orelabrutinib monotherapy.

Date	Nov 13 2025	Nov 19 2025	Dec 9 2025	Jan 19 2026	Feb 23 2026	May 7 2026	Normal range
APTT (s)	36.00	36.60	33.80	37.40	37.40	30.20	25.40-38.40
FVIII:C (%)	50.00	49.00	57.70	57.70	50.10	83.10	50.00-150.00
FVIII Inhibitor (BU)	2.20	3.20	1.40	<0.60	<0.60	<0.60	<0.60
Clinical Status	Relapse	Treatment initiated	Early response	CR2 achieved	Sustained CR	Sustained CR (on-treatment)	–

**Figure 1:**
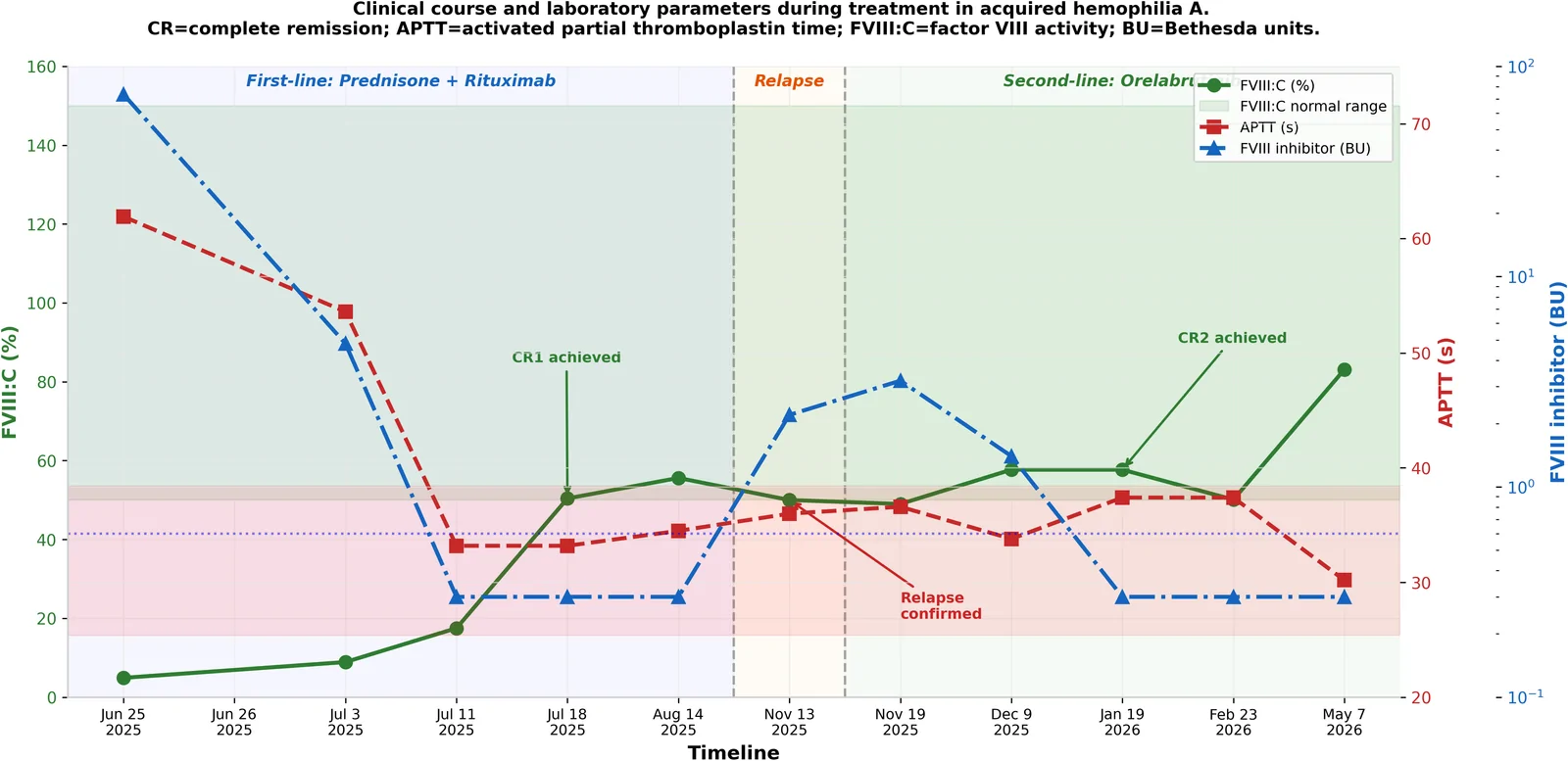
Clinical course and laboratory parameters during treatment in acquired hemophilia A. CR, complete remission; APTT, activated partial thromboplastin time; FVIII:C, factor VIII activity; BU, Bethesda units. The left portion of the figure (blue shading) shows the first-line treatment phase with prednisone and rituximab, during which CR1 was achieved. The middle portion (orange shading) shows the relapse period. The right portion (green shading) shows the second-line treatment with orelabrutinib monotherapy, with progressive improvement and achievement of CR2.

## 3 Discussion

This case represents the first reported use of orelabrutinib, a selective covalent BTK inhibitor, as monotherapy for AHA that relapsed after an initial response to conventional immunosuppression. The patient achieved rapid and sustained CR with complete inhibitor eradication and FVIII activity normalization, without requiring corticosteroids or cytotoxic agents. This observation has several important implications for AHA management.

AHA management requires a dual approach addressing both immediate hemostasis and long-term inhibitor eradication (2, 3). Current guidelines recommend corticosteroids as first-line immunosuppressive therapy, with addition of rituximab for enhanced response rates (3). While 60-80% of patients achieve CR with first-line therapy, relapse remains a significant concern, affecting 15-33% of responders, typically occurring within weeks to months after treatment discontinuation (5). The pathophysiology of relapse likely involves incomplete depletion of autoreactive B-cell clones and persistence of long-lived plasma cells secreting anti-FVIII antibodies (1).

Management of relapsed AHA presents several challenges. Re-treatment with corticosteroids carries cumulative toxicity risks, particularly in elderly patients with comorbidities such as diabetes (1, 6). Rituximab re-exposure may be less effective due to residual B-cell depletion or development of resistance. Alternative agents including cyclophosphamide, bortezomib (proteasome inhibitor), or calcineurin inhibitors have been explored in refractory cases, but evidence remains limited to small case series (7). There is a clear unmet need for targeted, less toxic therapies capable of achieving durable remission in relapsed AHA. Recent real-world data have likewise underscored the prognostic significance of early inhibitor eradication and the persistent burden of relapse in AHA (20). In our patient, the authenticity of the relapse was supported by supplementary testing: immunofixation electrophoresis excluded an interfering monoclonal protein, and lupus anticoagulant testing was negative, thereby excluding assay-related confounding of the APTT-based measurements.

BTK plays a critical role in BCR signaling, regulating B-cell development, activation, proliferation, and survival (8, 9). In autoimmune disorders, BTK-dependent signaling pathways contribute to the pathogenesis of autoantibody production through multiple mechanisms (1): activation and maturation of autoreactive B cells (2); germinal center reactions generating high-affinity autoantibodies (3); class-switch recombination and somatic hypermutation; and (4) survival signals for antibody-producing plasma cells (**Figure 2**).

**Figure 2:**
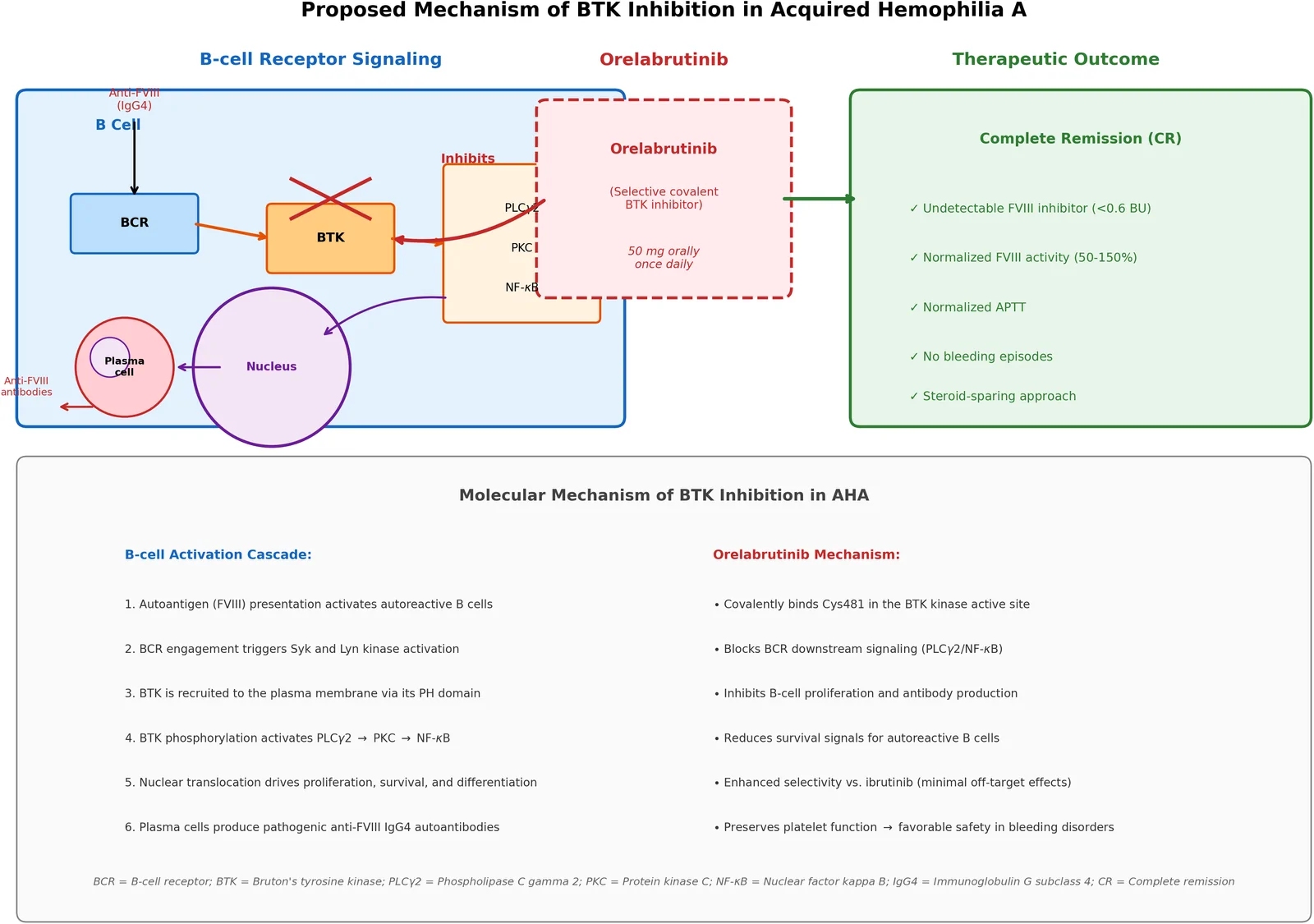
Proposed mechanism of BTK inhibition in acquired hemophilia A. (Left panel) Schematic illustration of BTK-dependent B-cell activation pathways contributing to anti-FVIII autoantibody production. BCR engagement by autoantigen (FVIII) triggers downstream signaling through BTK, PLC gamma 2, PKC, and NF-kappa B, leading to B-cell proliferation, differentiation into plasma cells, and production of pathogenic anti-FVIII IgG4 autoantibodies. (Center panel) Orelabrutinib, a selective covalent BTK inhibitor, binds Cys481 in the BTK kinase active site, blocking downstream signaling. (Right panel) Therapeutic outcomes of BTK inhibition include complete remission with undetectable inhibitor, normalized FVIII activity, and absence of bleeding episodes. BCR, B-cell receptor; BTK, Bruton’s tyrosine kinase; PLC gamma 2, phospholipase C gamma 2; PKC, protein kinase C; NF-kappa B, nuclear factor kappa B; IgG4, immunoglobulin G subclass 4; CR, complete remission.

BTK inhibitors have demonstrated efficacy across diverse antibody-mediated hematologic autoimmune disorders. In ITP, the oral reversible BTK inhibitor rilzabrutinib achieved platelet responses (platelet count of at least 50 x 10^9^/L) in 40% (24 of 60) of heavily pretreated patients in an open-label, dose-finding phase 1–2 trial (19). Ibrutinib has shown activity in acquired hemophilia and acquired von Willebrand disease associated with B-cell lymphoproliferative disorders (13, 14). These observations establish proof-of-concept for BTK inhibition in autoantibody-mediated coagulation factor deficiencies.

Orelabrutinib offers several pharmacological advantages over first-generation BTK inhibitors. Its enhanced kinase selectivity profile minimizes off-target inhibition of EGFR, ITK, and other Tec family kinases, translating to reduced toxicities (15). Specifically, the lower incidence of atrial fibrillation, bleeding events, and diarrhea compared to ibrutinib makes orelabrutinib particularly attractive for AHA patients who are already at increased bleeding risk. The once-daily oral dosing also improves treatment adherence.

The rapid inhibitor eradication observed in our patient (from 3.20 BU to <0.60 BU within approximately 2 months) is consistent with suppression of the autoreactive B-cell clone responsible for anti-FVIII antibody production, although mechanistic investigations such as B-cell subset or clonality analyses were not performed in this single-patient study. The sustained CR during continued therapy indicates that maintenance BTK inhibition may prevent resurgence of pathogenic B-cell populations. This is consistent with the mechanism of BTK inhibitors in other autoimmune disorders, where continuous therapy is required to maintain immune modulation (9, 10).

Importantly, our patient achieved CR without concurrent corticosteroids, representing a truly steroid-sparing approach. This is clinically significant given the substantial morbidity associated with prolonged corticosteroid exposure, including infection risk, metabolic derangements, osteoporosis, and cardiovascular events (1) - particularly relevant in AHA’s typical elderly population and in our patient with type 1 diabetes mellitus.

The absence of bleeding complications during orelabrutinib therapy is noteworthy. While non-selective BTK inhibitors such as ibrutinib are associated with increased bleeding risk through platelet function inhibition via glycoprotein VI and integrin alpha-IIb beta-3 pathways (8–10), orelabrutinib’s improved selectivity may mitigate this concern (15). In our patient with AHA - a disorder inherently characterized by bleeding diathesis - the lack of treatment-emergent hemorrhage supports the safety of selective BTK inhibition in this population.

We acknowledge several limitations of this report. First, as a single-case observation, these findings require validation in larger cohorts. Second, both remissions reported here were achieved on treatment, and our follow-up on orelabrutinib was limited to approximately 5 months. Given that 15-33% of AHA remissions relapse within months of immunosuppression cessation, longer-term observation is required to determine whether selective BTK inhibition can deliver sustained, treatment-free remission; encouragingly, remission has so far been maintained approximately 3 months after orelabrutinib discontinuation. Third, the optimal duration of orelabrutinib therapy in AHA remains undefined - whether treatment can be safely discontinued after sustained CR (as with conventional immunosuppression) or requires indefinite maintenance (analogous to autoimmune disorders requiring chronic immune modulation) warrants investigation. Fourth, the genetic predisposition suggested by the familial occurrence in this AHA case deserves further exploration through genetic studies.

In conclusion, we report the first case of relapsed AHA successfully treated with orelabrutinib monotherapy, achieving rapid and sustained CR with complete inhibitor eradication and FVIII activity normalization. This observation supports the therapeutic potential of selective BTK inhibition as a targeted, steroid-sparing strategy in relapsed or refractory AHA. Given the limited treatment options and significant relapse rates with conventional immunosuppression, BTK inhibitors represent a promising novel therapeutic class warranting prospective clinical investigation in AHA management. Longer-term follow-up after orelabrutinib discontinuation will be essential to establish the durability of treatment-free remission.

## Data Availability

The original contributions presented in the study are included in the article/supplementary material. Further inquiries can be directed to the corresponding author.
